# Biological activities and application of *Rosmarinus officinalis* extract to improve the preservation and microbial qualities of some local meat products

**DOI:** 10.1038/s41598-025-14247-x

**Published:** 2025-08-21

**Authors:** Basma T. Abd-Elhalim, Esmat S. Mohamed, Gamar Mahamat Gamar, Hanan Moawad

**Affiliations:** 1 Department of Agriculture Microbiology, Faculty of Agriculture, Ain Shams University, Hadayek Shoubra, PO Box 68, Shubra El-Khaimah, Cairo, 11241 Egypt; 2 Biotechnology Department, Higher Institute for Agriculture Co-Operation, Shubra, Cairo, Egypt; 3 Department of Life and Earth Sciences, Higher Institute for Teachers’ Training, P: 460, N’Djamena, Chad; 4 Plant department, Faculty of Science, Fayoum University, Fayoum, Egypt; 5 Department of Biology, College of Science, Jazan University, Jazan, 45142 Kingdom of Saudi Arabia

**Keywords:** Biofilm, Local meat products, *Rosmarinus officinalis*, Spoilage pathogenic, Natural antimicrobials, Microbiology, Antimicrobials, Applied microbiology, Biofilms, Industrial microbiology, Pathogens

## Abstract

Because medicinal plants contain bioactive phenolic compounds with antibacterial, antioxidant, and other properties, they have been used in many parts of nutrition and healthcare. The atmosphere in which local meat products and vendors operate is still subpar. Every individual is impacted, either directly or indirectly, when food is contaminated by harmful germs and spoilage. In this study, the microbiological purity, preservation, and shelf life of beef meat were assessed. The study analyzed the bacterial count in beef burger and luncheon samples, with gram negative bacteria having the highest contamination rate (58%). Four selected bacteria isolates were identified based on their morphological and cultural characteristics, confirmed by VITEK 2 system analysis and were identified as bacterial isolates from the Bacillaceae, Enterobacteriaceae, Pseudomonadaceae, and Staphylococcaceae families. The study found nine active compounds in rosemary leaves extract, including ferruginol, camphor, cineole, verbenone, borneol, αcaryophyllene, terpinen-4-ol, 2-methyl-4-vinylphenol, and eugenol, which act as antioxidants and antibacterials. The aqueous rosemary leaves extract (ARLE) showed efficacy in antibacterial activity against various bacteria, with clear inhibition zones (CIZ) and mean growth inhibition (MGI) values of 28.1 mm and 37%, respectively. The addition of ARLE to meat beef significantly reduced counts of pathogenic bacteria during storage. The preservation effectiveness of ARLE against artificially inoculated *Staphylococcus aureus* and *Pseudomonas aeruginosa* in meat beef exhibited complete bactericidal effect, with no recovery of the pathogen. The study assessed the impact of ARLE on meat beef sensory quality, finding that at a concentration of 15 mg/g, ARLE did not affect overall acceptability but enhanced the meat’s sensory properties. When used as a natural antioxidant agent, the ARLE’s antioxidant activity through DPPH scavenging proved to be successful in food preservation. Its radical-scavenging activity increased with concentration, with high DPPH radical-scavenging activity at 2560 μg/mL and varying percentages at different concentrations. ARLE also showed cytotoxicity against colon carcinoma cells (HCT-116), breast carcinoma cells (MCF-7), and human hepatocellular carcinoma cells (HepG-2) for 24 h. Applying ARLE significantly extended meat shelf-life by reducing microbial counts and inhibiting pathogens. To strengthen this conclusion, referencing specific safety and spoilage thresholds, such as TVC below 10^^6^ CFU/g, would provide a clearer, quantitative assessment of preservation. Demonstrating that treated samples remained below these limits longer than controls would offer concrete evidence of practical shelf-life extension.

## Introduction

Simply by virtue of eating, everyone runs the risk of being ill from a foodborne infection. According to the World Health Organization (WHO), the safety of food is the guarantee that, when prepared and/or consumed in line with its intended usage, food will not cause damage to the consumer. Additionally, according to^1^, hygiene of food refers to all the precautions taken to guarantee the safety, soundness, and wholesomeness of food during its whole production or manufacturing process as well as during ultimate consumption. Foodborne infections are responsible for millions of diseases worldwide each year, following the Centers for Disease Control and Prevention (CDC). Human cases of Salmonellosis are most likely caused by tainted meat and dairy products globally^2,3^. The microbiological quality of food has become a major global problem, and food-associated illnesses caused by food-poisoning bacteria have serious negative impacts on individuals and the economy. The prevalence of foodborne infections remains high in both the general population and at-risk groups, including as infants and young children, the elderly, and people with compromised immune systems^4,5^. Foodborne diseases, or FBDs, are a global problem that affects people on a daily basis, regardless of their level of development. Foodborne pathogenic microbes can cause diseases, hospitalizations, fatalities, and financial losses when consumed in conjunction with their toxins. According to reports, up to 30% of people in developed nations suffer from FBDs annually^6,7^. In the last two decades, the epidemiology of foodborne illnesses has changed significantly, with a rise in the incidence of bacterial infections caused by recently identified species as well as illnesses from well-known pathogens like *Salmonella* sp.^8^. Of food-borne illnesses requiring hospitalization, 60% include bacteria as a prevalent cause. Within the group of pathogens listed by ^1^, *Shigella* sp., *Campylobacter* sp., and *Salmonella* sp. are ubiquitous in nature and cause the majority of foodborne disease cases. According to ^9^, *Salmonella* sp. is the primary cause of 31% food-related fatalities, with *Listeria* sp. coming in second with 28%, *Campylobacter* sp. with 5%, and *Escherichia coli* and 3%. These bacteria increase the risk of foodborne illness. Additionally, *Pseudomonas aeruginosa*, *Bacillus subtilis*, *Lactobacillus* sp., *Saccharomyces cerevisiae*, and *Aspergillus niger* are food spoilage microbes that cause financial losses^9–11^. The research of next-generation food packaging materials derived from naturally occurring plants with antibacterial qualities has received more attention lately. Due to consumer demand for food products free of chemical preservatives, food manufacturers are now using organically produced antibacterial agents instead of traditional chemical ones^12^. There are several advantages to using natural plants as antimicrobial agents: first, they are cheap, particularly in developing nations where access to pricey western medications is limited; second, natural spices free of chemical synthetic products should be safer and have fewer adverse effects; and third, they are free of harmful effects on the environment. Therefore, using these natural plant extracts for food preservation to increase product shelf life is both feasible and practical^13,14^. In particular, plant extracts’ antioxidative and antibacterial properties have served as the foundation for a wide range of uses, including the preservation of both raw and processed food^15^. Natural antioxidants are receiving more attention due to the potential toxicity of synthetic antioxidants such as butylate hydroxyanisole (BHA) and butylated hydroxyl-toluene (BHT)^16^.

This study set out to determine how well rosemary aqueous leaf extracts inhibited pathogenic and contaminating microorganisms. This might serve as a viable substitute for the usage of conventional antimicrobials in food preservation. Moreover, the investigation of antioxidants as a scavenger of free radicals, different ant virulence, and anti-tumor activity was conducted.

## Results

### Microbiological assessment

#### Total aerobic bacterial count

The data obtained revealed that 50 beef burger samples contained the highest record of total bacterial count of 64% with an average of 4.24 log. Luncheon samples contained the lowest 18% with an average of 5.48 log (Table 1).

**Table 1:** Assessment of total aerobic bacterial counts of meat products.

Total aerobic bacterial counts			Number of food samples
Average (log 10)	Incidence %	Contaminated samples	
4.24	64	32	Beef burger (n = 50)
3.48	36	18	Luncheon (n = 50)

#### Incidence of gram positive bacteria (GPB)

Incidence of* B. cereus, L. monocytogenes,* and* S. aureus* was found in 25 out of 32 beef burger samples (9.3, 18.8%, and 2.43, 1.4, and 3.76 Log), and in 18 luncheon samples (16, 7, 11.1, and 16.7%, respectively, with 2.25, 2.65, and 2.85 Log) (Table 2). The results showed that GNB had a larger incidence of food contamination than GPB, with a higher count number log 18.74 and a higher incidence of 58%, whereas GPB had the lowest incidence log 15.34 and the highest count number log 18.74 (Table 2).

**Table 2:** Morphological and cultural characteristics of the isolated bacteria.

Test	*B. cereus*	*S. aureus*	*P. aeruginosa*	*E. coli*
Shape of colony	Flat	Raised, circular, entire	Flat	Low convex, entire
Texture	Rigid	Smooth	Smooth	Smooth
Pigmentation	Clear	Goldy yellow	Blue greenish	Clear
Motility	+	-	+	+
Oxygen requirements	Aerobic	Aerobic	Aerobic	Facultative aerobe
Gram reaction	G + ve	G + ve	G-ve	G-ve
Cell shape	Rods single or pairs	Cocci in clusters	Short single rods	Short single rods
Sporulation	+	-	-	-
Capsule	-	-	-	-
Coagulase	+	+	-	-
Hemolysis on blood agar	β	β	β	γ

*F* facultative, *(* +*)* positive, *(-)* negative.

#### Identification of the isolated bacteria

The high frequency and prevalence of bacteria in meat products led to the identification of four different bacterial isolates based on their morphological and cultural characteristics, which the VITEK 2 system analytical technique confirmed. The following were the outcomes of the identifying process:

#### Morphological and cultural characteristics

According to their morphological and cultural traits, the four detected bacterial isolates, *B. cereus, E. coli, P. aeruginosa,* and* S. aureus*, belonged to the four main bacterial families Bacillaceae, Enterobacteriaceae, Pseudomonadaceae, and Staphylococcaceae (Table 2).

#### *B. cereus* identification

After being separated*, B. cereus* was counted on plates containing *B. cereus* agar supplemented with egg yolk and Polymyxin B. Precipitation zone encircling the alleged colonies. The complete biochemical identification of* B. cereus* using the VITEK 2 technology resulted in an exceptional likelihood of 99.35% for its biochemical properties. According to the VITEK 2 system, the antibiotic sensitivity of *B. cereus* demonstrated a range of susceptibilities against several tested antibiotics, including sensitive, intermediate, and resistant.

#### *E. coli* identification

After isolation,* E. coli* was counted on Eosin Methylene Blue (EMB) plates. Once* E. coli* was fully identified biochemically using the VITEK 2 technology, its biochemical properties were verified with a good probability of 99%. According to the VITEK 2 system, *E. coli* antibiotic sensitivity varied, showing sensitivities to several tested antibiotics that ranged from sensitive to intermediate to resistant.

#### *P. aeruginosa* identification

*P. aeruginosa* colonies was streaked on cetrimide agar plates—a selective medium that increases the synthesis of the "blue-green" pigments pyocyanin and fluorescein—confirmed these colonies. Following complete biochemical identification using the VITEK 2 technology, the biochemical features of *P. aeruginosa* were verified with a good probability of 99.75%. According to the VITEK 2 system, *P. aeruginosa* antibiotic susceptibility ranged from sensitive, intermediate, and resistant to several tested antibiotics.

#### *S. aureus* identification

*S. aureus* was typically isolated and counted onto blood medium plates. ViTIK2’s complete biochemical identification of *S. aureus* allowed for the great likelihood (99%) confirmation of its biochemical properties. According to the VITEK 2 system, *S. aureus* antibiotic sensitivity varied, ranging from intermediate to resistant against several tested antibiotics.

### Active ingredient compounds of rosemary leaves extract (RLE)

Nine compounds were found to be active in the RLE, and upon analysis, it was determined that their chemical formula and determinate groups acted as antioxidants and antibacterials: ± caryophyllene, terpinen-4-ol, 2-methyl-4-vinylphenol, eugenol, and camphor (3.88%, 4.53%, 2.66%, verbenone, 2.50%, & borneol, 1.56%) (Table 3).

**Table 3:** Phytochemical compounds of rosemary leaves extract (RLE).

Retention time	Compounds	Molecular formula	Molecular weight	Area (%)
8.80	Cineole	C_10_H_18_O	154	2.66
11.71	Caryophyllene	C_15_H_24_	204	0.40
13.36	Camphor	C_10_H_16_O	152	3.88
14.44	Borneol	C_10_H_18_O	154	1.56
14.88	Terpinen-4-ol	C_10_H_18_O	154	0.25
16.26	Verbenone	C_10_H_14_O	150	2.50
21.09	2-methyl-4 vinylphenol	C_9_H_10_O	134	0.15
22.85	Eugenol	C_10_H_12_O_2_	164	0.09
58.70	Ferruginol	C_20_H_30_O	286	4.53

### Bioactivity of ARLE as antibacterial activity

The clear inhibition zones (CIZ) and mean growth inhibition (MGI) data demonstrated the effectiveness of ARLE’s antibacterial action against isolates of bacteria. The results showed 37% MIG and 28.1 mm CIZ of *B. cereus*. There were 18.2 mm CIZ and 94.4% MIG of *E. coli*. *P. aeruginosa* showed 92% MIG and 26.8 mm CIZ. *S. aureus* had 35% MIG and 16 mm CIZ. (Table 4).

**Table 4:** The antibacterial activity of ARLE (100 mg/mL/disc) related to Ampicillin and Gentamicin based on the growth inhibition and disc diffusion method of bacterial isolates.

Bacterial isolates Treatments		*B. cereus*	*S. aureus*	*E. coli*	*P. aeruginosa*
ARLE	IZ mm	28^a^ ± 0.54	26^a,b^ ± 0.94	9.2f. ± 0.88	11.8^e^ ± 0.69
	%MGI	37 ± 0.56	35 ± 0.56	94.4 ± 0.71	92 ± 0.82
Ampicillin	IZ mm	18^c^ ± 0.56	15^d^ ± 0.56	0.00	0.00
	%MGI	87 ± 0.56	75 ± 0.56	0.00	0.00
Gentamicin	IZ mm	0.00	0.00	0.00	0.00
	%MGI	0.00	0.00	64.4 ± 0.71	72 ± 0.82

*IZ mm* Inhibition Zone diameter. (%) Mean growth inhibition percentage, % MGI = Mean growth inhibition percentage (O.D.620). Each value is mean by 3 replicates ± standard error. Values with the same letter do not differ significantly from each other, according to^17^, at a 5% level.

### Minimum inhibitory concentrations (MIC) of ARLE

MIC of ARLE was 0.475, 1.125, 1.225 and 0.875 mg/mL for *B. cereus, E. coli, P. aeruginosa* and *S. aureus*, respectively.

### Antivirulence activity of ARLE

#### Biofilm formation of bacteria isolates

Biofilm is a virulent factor for survival of bacteria in various environments including food products. Biofilm formation was classified as strong (0.7–1.0), moderate (0.3–0.4) and weak biofilm ≥ 0.3 (O.D. 570). Four isolated bacteria formed positive biofilm phenotypes 1.12, 1.25, 0.69 and 0.75 (O.D. 570) of *B. cereus, E. coli, P. aeruginosa* and *S. aureus*, respectively.

#### Biofilm inhibition

At sub-MICs of 0.0, 3.00, 1.50, 0.750, 0.375, and 0.184 mg/mL, the ARLE examined its ability to inhibit the development of biofilms in bacterial isolates. This resulted in decreased biofilm formation with 75.4, 58.5, 36.0, and 0% *E. coli*; 80.0, 60.8, 58.4, 51.2, & 45.6% *P. aeruginosa*; 97.1, 87.0, 82.6, 72.5, & 69.5% *B. cereus*; and 95.3, 90.7, 78.8, and 76.5% *S. aureus*, in that order. (Table 5).

**Table 5:** Biofilm and anti-biofilm formation of tested pathogenic bacteria isolates treated with Sub (MICs) concentrations of ARLE.

Pathogenic bacteria isolates		Sub (MICs) concentrations mg/mL					
		0.00	3.00	1.50	0.750	0.375	0.184
Biofilm formation O.D	*E. coli*	1.12^a^	0.32^i^	0.54f.	0.62^e^	0.68^d^	0.72^c^
	*P. aeruginosa*	1.25^a^	0.25^j^	0.49^ h^	0.52^ fg^	0.61^e^	0.68^d^
	*B. cereus*	0.69^d^	0.02^o^	0.09^n^	0.12^ m^	0.19^kl^	0.21^ k^
	*S. aureus*	0.85^b^	0.04^o^	0.08^n^	0.13^ m^	0.18^ l^	0.20^ k^
Biofilm reduction (%)	*E.coli*	0 0.0	71.4	51.8	44.6	44.0	35.7
	*P. aeruginosa*	0 0.0	80.0	60.8	58.4	51.2	45.6
	*B. cereus*	0 0.0	97.1	87.0	82.6	72.5	69.5
	*S. aureus*	0 0.0	95.3	90.6	84.7	78.8	76.5

Values with the same letter do not significantly differ from each other, according to^17^, at a 5% level.

### Antihemolytic activity of ARLE against *S. aureus*

The results showed the hemolysis optical density of human erythrocytes by *S. aureus* culture supernatant. The optical density of hemolysis human erythrocytes (RBCs) by *S. aureus* culture was 0.432 OD. While the optical density of RBCs by *S. aureus* culture treated with sub (MICs) 0.781, 0.390, 0.195 and 0.097 (mg/mL) ARLE were 0.042, 0.074, 0.178 and 0.296 O.D with strong hemolysis activity reduction 92.35, 90.76, 47.13 and 13.37% respectively (Table 6).

**Table 6:** Antihemolytic effect of ARLE against *S. aureus.*

Antihemolytic activity		Sub MICs (mg/mL)				
		0.000	0.781	0.390	0.195	0.097
Hemolysis	Activity O.D	0.432^a^	0.042^e^	0.074^d^	0.178^c^	0.296^b^
	% Reduction	0.000	90.28	82.87	58.79	31.48

Values with the same letter do not significantly differ from each other, according to^17^, at a 5% level.

### Investigation of treated and non-treated bacteria via TEM

**Fig. 1:**
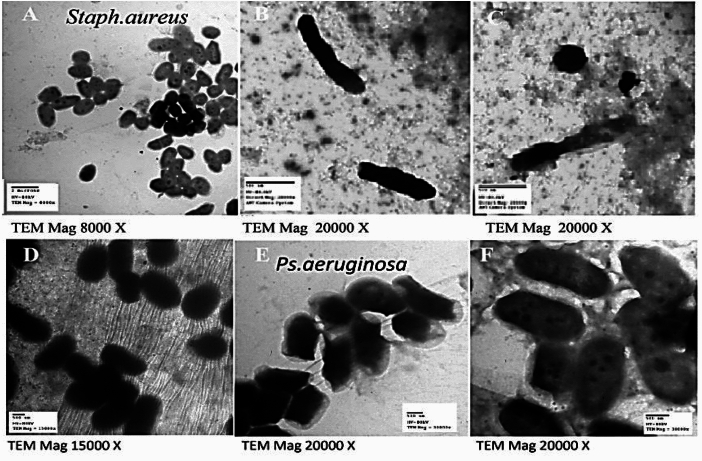
TEM images showing morphologically damaged cells of *P*. *aeruginosa* and *S*. *aureus* treated with ARLE (**B**, **C**, **E** & **F**) and images of morphological normal non-treated cells (**A** & **D**).

The TEM images presented in Fig. 1 illustrate the morphological effects of the ARLE on bacterial cells. Specifically, the images compare treated and untreated cells of *P. aeruginosa* and *S. aureus*. The Fig. 1 labeled B, C, E, and F depict cells that exhibit morphological damage after exposure to ARLE, indicating disruption of cellular integrity. Such damage may include cell membrane disintegration, cell wall alterations, or internal cell content leakage, suggesting the antimicrobial activity of the extract. Conversely, the untreated cells shown in Fig. 1A,D display normal cell morphology, maintaining intact structural features. These TEM results highlight the efficacy of ARLE in damaging bacterial cells at a structural level, supporting its potential as an antimicrobial agent.

### Application of ARLE on preservation of meat

Due to a reduction in harmful bacteria to nil throughout the storage period and a longer storage period than the control, ARLE was added to beef (Table 6). The total aerobic bacterial, *S. aureus* and *P. aeruginosa* counts in meet beef samples incorporated with ARLE at concentrations 6.25 and 12.5 (mg/mL) and non-treated samples as a control were examined during entire storage period, 0 to 21 days at 4 °C. The results presented in (Table 6) showed that the total aerobic bacterial counts in control samples gradually increased from 3.66 to 13.78 CFU/g. On the contrary the count gradually decreased to zero number at the end of the storage period (21 days) (Table 6). The total counts of *S. aureus* and *P. aeruginosa* counts in beef incorporated with ARLE at a concentration 12.5 (mg/mL), were significantly reduced by 100% at 2^nd^, the 4^th^, the 6^th^ and the 8^th^ weeks of storage, which no visual colonies appeared after aerobic incubation for 24 h at 37 °C throughout the entire storage period compared to non-treated meat beef as a control samples were, 3.25, 4.22, 5.82, 6.15 , 8.72 and 11.21 and 13.78 CFU/g at the zero day, 2^nd^, 4^th^, the 6^th^ and the 8^th^ weeks of storage period respectively. These results clearly indicated that the preservation effectiveness of ARLE at minimum bactericidal concentration (MBC) of 12.5 (mg/mL) against artificially inoculated *S. aureus* and *P. aeruginosa* in meat beef exhibited complete bactericidal effect with no recovery of the pathogen (Table 7).

**Table 7:** Count of total bacteria, *P. aeruginosa* and *S. aureus* of beef samples treated with ARLE on different storage days.

Storage time (days)	Control (log 10 CFU/g)	Treated beef samples with ARLE (log CFU/g)					
		(6.25 mg/mL)			(12.5 mg/mL)		
		Total bacteria	*P. aeruginosa*	*S. aureus*	Total bacteria	*P. aeruginosa*	*S. aureus*
0	3.25 ± 0.28^ g^	3.20 ± 1.22^ g^	0.86 ± 0.51^ k^	0.75 ± 0.6^ l^	3.12 ± 1.12^ g^	0.65 ± 0.61^ m^	0.62 ± 0.61^n^
3	4.22 ± 1.32f.	2.25 ± 0.89^ h^	0.00 ± 0.00	0.00 ± 0.0	2.15 ± 0.49^ h^	0.00 ± 0.00	0.00 ± 0.00
6	5.82 ± 1.26^e^	2.00 ± 1.61^i^	0.00 ± 0.00	0.00 ± 0.0	1.92 ± 1.31^j^	0.00 ± 0.00	0.00 ± 0.00
9	6.15 ± 1.45^d^	2.12 ± 0.99^ h^	0.00 ± 0.00	0.00 ± 0.0	2.00 ± 0.69^i^	0.00 ± 0.00	0.00 ± 0.00
12	8.72 ± 1.48^c^	2.18 ± 1.11^ h^	0.00 ± 0.00	0.00 ± 0.0	2.08 ± 1.00^i^	0.00 ± 0.00	0.00 ± 0.00
18	11.21 ± 1.15^b^	2.05 ± 1.35^i^	0.00 ± 0.00	0.00 ± 0.0	2.05 ± 1.15^i^	0.00 ± 0.00	0.00 ± 0.00
21	13.78 ± 1.61^a^	2.00 ± 1.33^i^	0.00 ± 0.00	0.00 ± 0.0	1.92 ± 1.10^j^	0.00 ± 0.00	0.00 ± 0.00

The mean standard error of the experiment’s three replications is represented by each reported number for the viable bacterium count. Values with the same letter do not significantly differ from each other, according to ^17^, at a 5% level.

### Organoleptic evaluation of meat beef

An organoleptic test was performed by ten panelists on meat beef treated with ARLE 15 and 30 mg/g meat beef to assess the impact on sensory quality. When compared to the control sample, there were no discernible variations in any of the qualities, with the exception of taste. At a dose of 15 mg/g, ARLE had no detrimental impacts on overall acceptability, but it did improve the taste of beef meat. (Table 8). ARLE improved the sensory properties of meat beef. It improves the pink-red color, shape, firm texture, and natural smell of the meat. In contrast, bacterial contamination changes the sensory properties of the meat to a dark red, yellow, and grey color, white spots on the meat surface, which are grey bacterial colonies in the form of small droplets, a sticky, moist texture, a loose texture, an unpleasant and pungent odor, a sulfuric ammoniac odor, and a sour taste.

**Table 8:** Sensory quality meat beef inoculated with *P. aeruginosa* and *S. aureus* and incorporated with ARLE.

Meat beef treatments		Organoleptic characteristics				
		Taste	Color	Texture	Odor	Overall acceptability
Control		4.75^a^	4.65^a^	4.85^a^	4.45^a^	4.35^a^
Bacteria contaminant		0.85^c^	1.40^c^	0.75^c^	1.15^c^	1.00^c^
Aquas rosemary leaves extract (ARLE)	15 mg/g	4.65^a^	4.55^a^	4.50^a^	4.75^a^	4.65^a^
	30 mg/g	3.25^b^	3.85^b^	2.75^b^	3.45^b^	3.15^b^

The sensory evaluation was conducted by five-point hedonic scale wherein: very poor = 0.5:1.5, poor = 1.5–2.5, common = 2.5–3.5, good = 3.5–4.5, very good = 4.5–5. Values with the same letter do not significantly differ from each other, according to^17^, at a 5% level.

### The maximum non-toxic concentration of ARLE

The maximum non-toxic dose (MNTD) of ARLE was imperative to determine Vero cell culture using cytotoxicity and MTT assay for studying the anticancer activity of ARLE. The MNTD was 250 μg/mL (mean of 3 replicates). It did not show any morphological difference in Vero cells when compared with control one.

### Antioxidant activity of ARLE

**Fig. 2:**
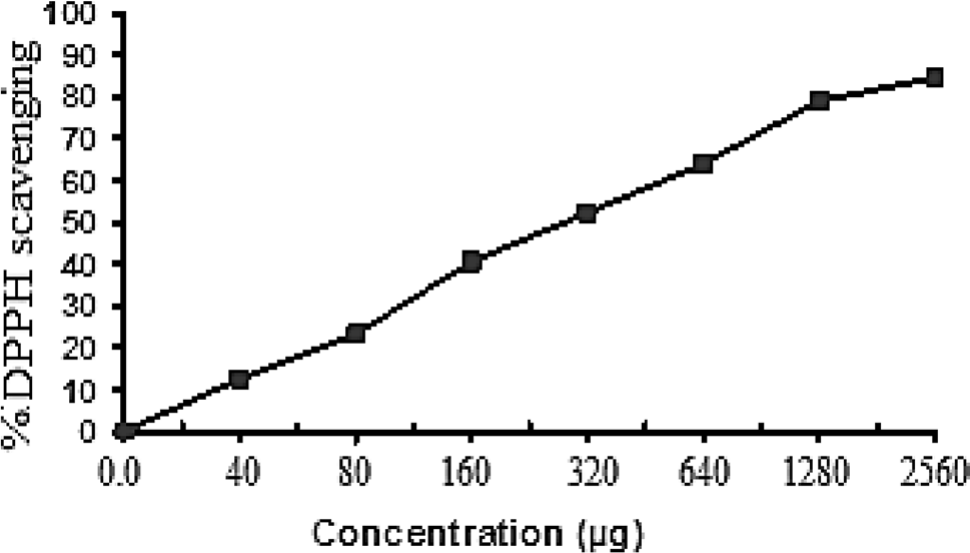
Curve of antioxidant activity of as determined by free radical-scavenging activity.

The assessment of ARLE’s overall antioxidant capacity through the utilization of the DPPH scavenging radical oxidation inhibition method, with the aim of ascertaining its antioxidant efficacy for potential application as a natural antioxidant agent in food preservation. Furthermore, by neutralizing the DPPH radical, ARLE demonstrated antioxidant activity in vitro. From the analysis of Fig. 2, As the concentration grew, the results showed that ARLE’s radical-scavenging activity increased as well. At 2560 µg/mL, it demonstrated a high level of DPPH radical-scavenging activity, with a scavenging activity of 84.46%. The data displayed the percentages of radical-scavenging (78.9, 63.92, 52.23, 40.54, 23.11, 12.50, and 0%) at different concentrations (1280, 640, 320, 160, 80, 40, and 0 µg/mL), with an IC_50_ value of 289.5 µg. The absorbance values were converted to scavenging effects (%) and data was plotted as the means of triplicate scavenging effect (%) values.

### Cytotoxicity against tumor cell lines

**Fig. 3:**
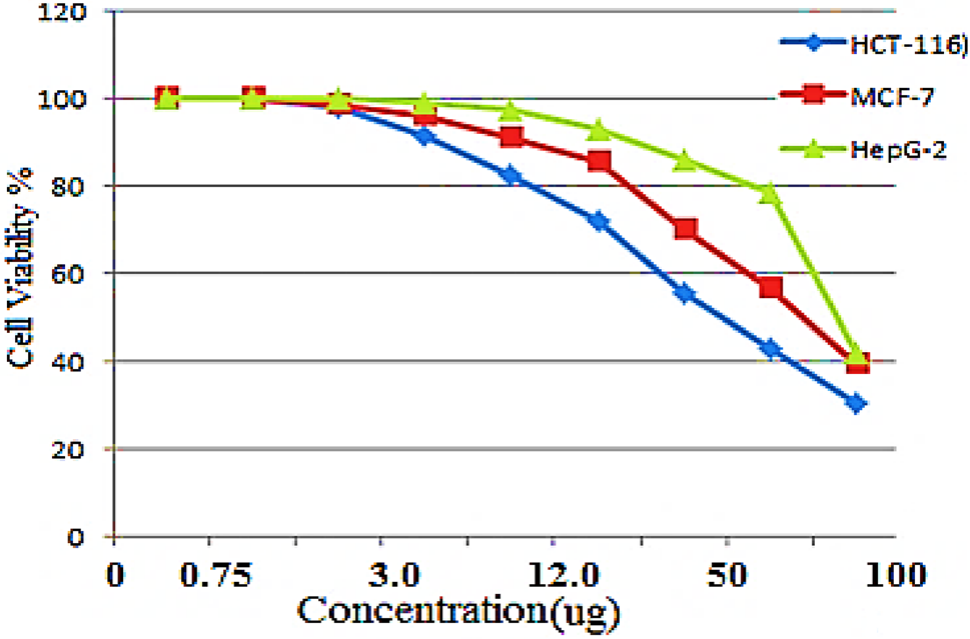
Curves showing the dose response of Tumor cell line (MCF-7 , HepG2 and HCT-116) after exposure to aqueous rosemary leave extract for 24 h.

**Fig. 4:**
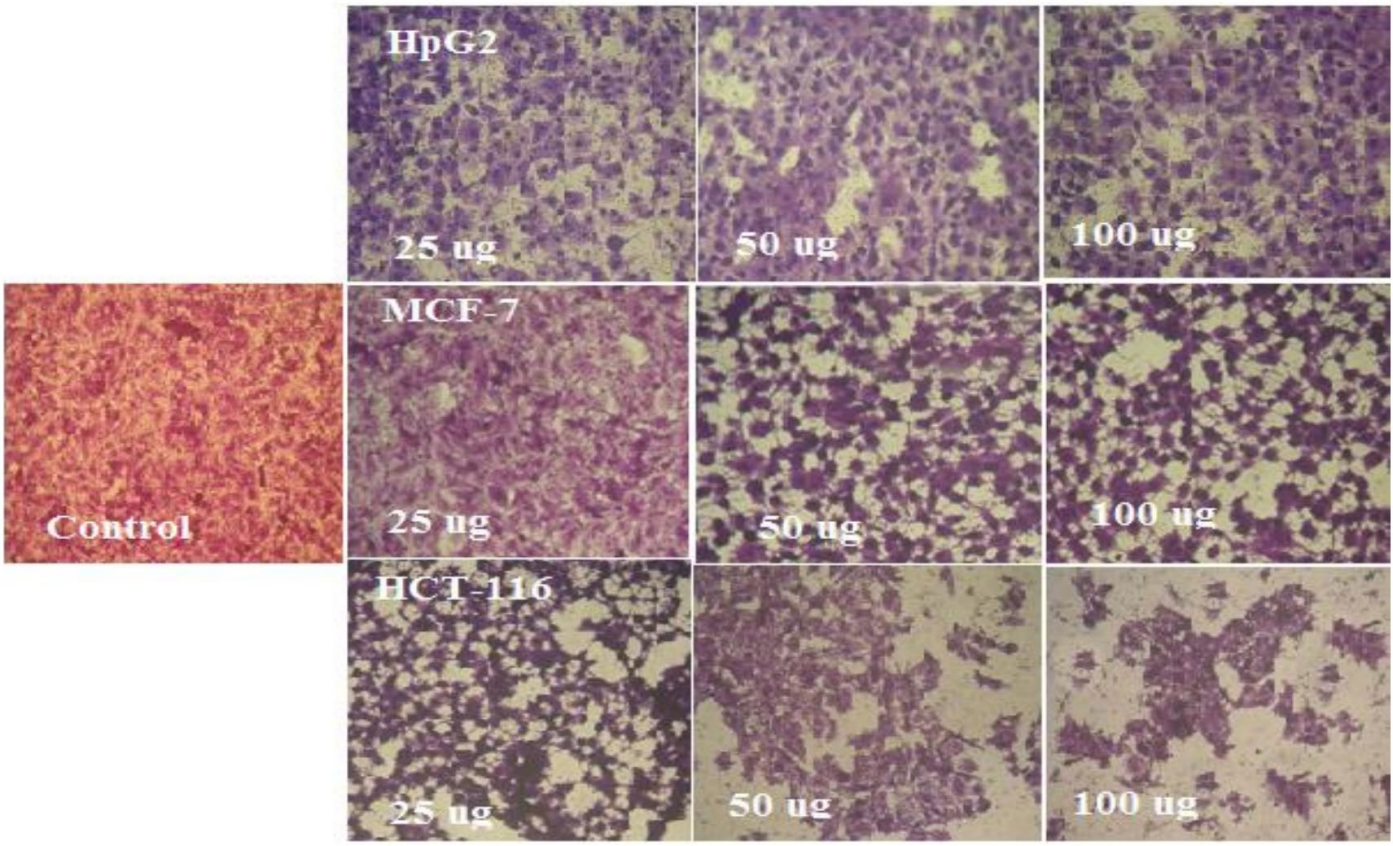
Photo images, showing effect of ARLE at different conc. (25, 50 and 100 ug) for 24 h on HepG-2, MCF-7 and HCT-116 cell-lines comparable to control.

Table 9 and Figs. 3 and 4 presented data on tumor cell lines colon cancer cells (HCT-116), breast carcinoma cell line (MCF-7) and human hepatocellular carcinoma cells (HepG-2) treated to ARLE (0 to 100 µg) 24 h, with cytotoxicity assessed. The HepG-2, MCF-7, and HCT-116 cell lines have 50% inhibitory concentration (IC_50_) values of 24.36, 62.24, and 74.12 µg, respectively.

**Table 9:** Antitumor activity of ARLE against tumor cell lines.

ARLE conc. (µg)	Tumor cell lines		
	HCT-116	MCF-7	HepG-2
0	100	100	100
0.75	100	100	100
1.50	97.25	98.45	100
3.00	91.75	95.24	98.74
6.00	82.44	91.74	96.25
12.0	73.12	86.32	92.45
24.0	54.74	72..33	86.54
48.0	42.23	62.23	77.35
100	26.42	45.12	45.23
IC_50_ (µg)	24.36	62.24	74.12

## Discussion

Food becomes dangerous for human consumption when it becomes contaminated or spoiled due to the addition of toxic ingredients or bacteria. We call this food contamination. The percentage of contamination-positive samples in the total aerobic bacterial count of the meat samples ranged from 28 to 86%, depending on the kind of meat product. These results agree with^18–23^, who found that 68% of samples had aerobic colony counts that exceeded 10^6^ CFU/g. Due to their exposure to room temperature, these meals may have allowed contaminating bacteria to proliferate, which in turn raised the number of germs. On the other hand, the meat products were contaminated with *S. aureus, B. cereus* and* L. monocytogenes*. Reference^24^ showed that incorrect handling, cross-contamination, and inadequate temperature control might be the cause of *S. aureus* found in ready-to-eat meals.

Our findings demonstrate that VITEK 2 systems were able to identify bacteria from meat and beef samples by morphological and biochemical testing. *E. coli*, *S. aureus*, *B. cereus*, and *P. aeruginosa*, were the most prevalent bacterial species; these findings were consistent with those found by ^25^.

The ARLE has been directly tested for its antimicrobial ability to reduce or prevent pathogenic and spoiling of gram positive and gram negative bacteria, making it a great alternative to the use of traditional antimicrobials in food preservation. Additionally examined were the various antivirulence, anti-carcinogenic, and free radical-scavenging characteristics of antioxidants.

Indeed, compared to their gram positive counterparts, gram negative bacteria show higher levels of antibiotic resistance. For instance, the process of membrane accumulation or the permeability barrier provided by the cell wall may cause resistance^26^. With inhibition zones that range in diameter from 17 to 24.8 mm, ARLE specifically exhibits a 100% mean growth inhibition percentage against the spectrum of tested bacteria and substantially effective growth inhibition on ten tested foodborne bacterial isolates. This trend of results agrees with^27^, They found that a variety of vegetable and plant extracts had excellent antibacterial activity with inhibition zones of 19.9 and 33 mm against *S. typhimurium* isolated from the poultry chain and *E. coli* ATCC 25,922.

The MIC is generally regarded as the most basic laboratory measurement of the activity of an antimicrobial agent against an organism^28^. Thus, determination of MICs has become the main factor for scientific studies regarding the feasibility of bioactive components of plants in industry^29^.

Based on our results most tested foodborne bacteria were sensitive in low concentrations to ARLE, *B. cereus*, had the lowest record (MIC) at 0.781 mg/mL for each bacterium, followed by *E. coli* and *S. aureus* at concentration of 1.562 mg/mL. The highest (MICs) values were recorded for *P. aeruginosa* at concentrations 6.25 mg/mL for both bacteria for total extract. These results are in harmony with^30^, who discovered that when clove aqueous extract, either pure or in combination with rosemary (*R. officinalis* spp.) aqueous extract, was tested against *S. epidermidis*, *S. aureus*, *B. cereus*, *E. coli*, *P. vulgaris*, and *P. aeruginosa*, the minimum inhibitory concentrations were found to be between 0.062% and 0.500% (v/v), which is encouraging for use as a food preservative or bacterium. Also, these results were agreed with^31^, who found that the oil of *Thymus vulgaris* had MICs of 0.04%, and 0.03%, respectively, against *L. monocytogenes* and* S. enteritedis.*

The food business and consumers are nevertheless worried about food poisoning and spoiling during manufacturing and storage, which are mostly brought on by oxidation processes or microbe activity, even with the use of artificial chemical additives. Some synthetic antioxidants that are used in food processing and preservation have carcinogenic effects in living organisms^32^. Thus, there is growing interest in the creation of novel, safe, and effective natural antioxidants. Numerous plant extracts are known to have antibacterial and antioxidant properties in food systems. Some herbs, like rosemary, may naturally create antioxidants. In many different dishes, Egyptians use rosemary leaves as a seasoning or flavoring. Nevertheless, the herb’s antioxidant capacity has not yet been fully explored and applied to improve food products’ shelf-stability. The total antioxidant activity of rosemary aqueous extract was evaluated using the radical DPPH oxidation inhibition approach to determine its antioxidant impact for use in food preservation. Additionally, the ARLE showed antioxidant activity in vitro by neutralizing the DPPH radical. The concentration that provided 50% inhibition (IC_50_) was used in this investigation to measure the whole extract’s ability to scavenge the free radicals DPPH and reduce power; a lower IC_50_ value indicates more radical-scavenging activity. The effective concentration of the sample in micrograms per milliliter (μg/mL) at which 50% of DPPH radicals are scavenged is known as the IC_50_, which is used to express antioxidant activity.

The results revealed IC_50_ of 289.5 μg/mL, these findings are in accordance with the antioxidative effect of thyme (*Thymus vulgaris*) observed by other authors^33–35^,who reported a high radical scavenging activities for *R. officinalis* by IC_50_ of 65.2 ± 0.3 and 85.5 ± 0.1 μg/mL respectively. The major constituent of Thyme is Luteolin, to which are attributed many of the antioxidant properties^36^.

According to the current study, the bacteria that formed the strongest biofilms were *E. coli*, *B. cereus*, *P. aeruginosa*, and *S. aureus*. These findings are consistent with the findings of^37^, who evaluated the microorganisms’ capacity to adhere to surfaces during food processing and form biofilms. As concluded by^38^ that *Bacillus* strains were often isolated from biofilms in the food industries.

Microorganisms form biofilms to protect themselves against adverse conditions. These Single-species or mixed-species biofilms are both possible, however mixed-species biofilms provide better protection due to their increased stability and ability to form a thicker, bigger mass of biofilm^39^. Following colonization and multiplication, the bacteria adhere to the surface to create a biofilm^40^. Microbial cells adhere to a moist surface and become fixed in a protective polysaccharide matrix to create an efficient group known as a biofilm. By trapping additional bacteria and nutrients, this matrix can encourage the growth of microorganisms. When it comes to sanitation agents, connected bacteria are more resilient than detached ones.

The extracellular polymeric substance (EPS) layer, which keeps chemicals out of the biofilm or renders the sanitizer ineffective, and protection from organic contaminants are the sources of this resistance^40^. It has been shown that a wide variety of food contact and non-contact surfaces can be adhered to by bacterial pathogens and spoiling agents. The financial performance of the food business may suffer as a result of this occurrence. Finding a natural, distinct biofilm inhibitor is therefore essential for food safety procedures and goods.

Research on biofilm inhibition with ARLE at all tested concentration levels (MIC) has effectively prevented the growth of biofilms in *E. coli*, *B. cereus*, *Pseudomonas* sp., and *S. aureus*, with percentages of biofilm reduction of 95.2, 75, 98.6, and 62.25, respectively. Because biofilms are naturally resistant to existing antibiotic medicines, biofilm infections are becoming an increasing challenge to contemporary medicine^41^. These findings are in the line with those of^42,43^, who reported on the antibiofilm activity of several plant extracts against human pathogenic bacterial biofilms. The matrix’s makeup changes depending on the kind of organism, and plant extract weakens the biofilm by reducing the biochemical content of the matrix^44^.

The majority of anticancer medications in use today are extremely costly, toxic, and problematic due to resistance mechanisms^45,46^. Spices and other food-adaptive components show their antimutagenic and anticarcinogenic effects in the following ways: (1) by scavenging DNA reactive agents, (2) by inhibiting carcinogen activation and enhancing carcinogen detoxification, (3) by suppressing the abnormal proliferation of early preneoplastic lesions, and (4) by altering certain characteristics of the cancer cell.

This work examined the cytotoxic effects of ARLE dietary adaptation on three cancer-derived cell lines: HepG-2, MCF-7, and HCT-116. The data obtained indicate that ARLE can generate cytotoxic activity against the three cell lines with IC_50_ values of 89, 69.9, and 36 µg, respectively. The extract’s IC_50_ value is the concentration at which, in comparison to untreated controls, there is a 50% reduction in total metabolic activity.

These results showed that the survival rates of the three cell lines usually declined in a dose-dependent way. The HCT-116 cell line, which is considered to have potential cytotoxicity, also showed higher sensitivity to ARLE in comparison to the other two cell lines. These findings are in trend with those of other scientists^47^, who found that carvacrol, a component of thyme extract, has an important anticancer impact on tumor cell lines like HepG-2.

Furthermore, these findings support the findings of ^48^ on the antioxidant and anticancer effects of thymol. Moreover, it has been suggested that thymol and carvacrol are the main substances in thyme and rosemary that are responsible for some of their pharmacological properties^47^.

Furthermore, natural substances that cause cancer cells to undergo apoptosis are useful instruments for preventing cancer. For extracts with therapeutic promise, there are two mechanisms: chemoprevention and cancer suppression. Some of the techniques used to cure cancer include antimetastatic, antiangiogenetic, and detoxifying enzyme activation, as well as the blocking of DNA repair signals. When compared to the sample control samples, the efficacy of ARLE application in preserving beef and increasing its shelf life at 6.25 mg/mL demonstrated a significant decrease in viable total bacterial counts and pathogenic bacteria during the whole storage period. These findings align with the findings of^47,48^. For meals to have the same in-vitro impact, a higher concentration of dried herbs, spices, or their extracts is often required.

The compounds identified in rosemary leaves extract, including ferruginol, camphor, cineole, verbenone, borneol, α-caryophyllene, terpinen-4-ol, 2-methyl-4-vinylphenol, and eugenol, exert their antibacterial, antioxidant, and cytotoxic effects through diverse and interconnected mechanisms at the molecular level. Regarding antibacterial activity, many of these compounds interact with bacterial cell membranes, disrupting their structural integrity and increasing permeability^7,48^. For example, cineole and eugenol are known to embed into lipid bilayers, causing fluidity changes that result in leakage of vital ions and cellular components. Monoterpenoids like camphor and borneol similarly insert into membranes, disturbing their function and impairing essential processes such as enzyme activity and energy production. Eugenol, in particular, can inhibit key bacterial enzymes involved in cell wall synthesis and ATP generation, weakening bacterial defenses and inhibiting growth. Additionally, some of these compounds may induce oxidative stress in bacteria by generating reactive oxygen species (ROS), further damaging cellular components and enhancing their antimicrobial efficacy. The antioxidant activities of these compounds are primarily due to their ability to neutralize free radicals and ROS that cause cellular damage. Phenolic compounds like ferruginol and eugenol possess hydroxyl groups capable of donating hydrogen atoms, effectively scavenging free radicals and halting radical chain reactions. Cineole and verbenone, though less reactive, can influence oxidative stress indirectly by upregulating the body’s endogenous antioxidant enzymes or chelating transition metals involved in ROS formation. These actions help protect cellular lipids, proteins, and DNA from oxidative damage, maintaining cellular integrity and function under stress conditions. In terms of cytotoxic and anticancer effects, these compounds can induce apoptosis and inhibit proliferation in cancer cells through multiple pathways. Ferruginol and eugenol have demonstrated the ability to cause mitochondrial dysfunction by disrupting mitochondrial membrane potential, leading to the release of cytochrome c and activation of apoptosis-related caspases. They can also generate elevated intracellular ROS levels, resulting in oxidative damage to critical cellular components and triggering programmed cell death. Moreover, certain compounds like terpinen-4-ol and camphor may modulate key signaling pathways such as NF-κB and MAPK, which regulate cell growth and survival, thereby inducing cell cycle arrest and apoptosis. These mechanisms collectively impair tumor growth and metastasis, making these compounds promising candidates for anticancer therapy. It is also important to recognize that these compounds often work synergistically. Their combined effects may enhance membrane disruption, boost antioxidant defenses, or amplify cytotoxic responses more than any single compound alone. For example, the membrane-disruptive actions of monoterpenoids facilitate better penetration of phenolic compounds like eugenol, intensifying their antimicrobial and anticancer effects. Furthermore, their antioxidant properties can help mitigate collateral tissue damage during therapeutic applications, reducing side effects and improving overall efficacy. In conclusion, the bioactivities of these rosemary-derived compounds are rooted in their ability to target multiple cellular processes. Their capacity to disrupt microbial membranes, neutralize oxidative stress, induce mitochondrial dysfunction, and modulate signaling pathways underpin their potent antimicrobial and anticancer properties. These multifaceted mechanisms highlight their potential as natural agents for improving food preservation and developing new therapeutic strategies.

## Methods

### Collection and preparation of meat products samples

During Jun to August in 2023, a total of 100 meat products were randomly collected from different markets at Cairo and Qalyubia governorates included 50 Beef burger with code Bb and 50 Luncheon with code L in plastic sterile bags, and transferred in ice-boxes to bacteriology Lab. Twenty-five grams of each samples was mixed with 225 mL buffered peptone water and homogenized in sterile mixer, to make sufficient dilutions for the microbiological analysis^49^. Ten-fold dilutions of homogenate samples were prepared in sterilized test tubes.

### Chemicals and media used

The study employed a variety of chemicals and media sourced from various suppliers. For microbiological analyses, Plate Count Agar (Oxoid, UK; product code CM325) was used to assess bacterial counts, while Tryptic Soy Broth with 2% glucose was obtained from Sigma-Aldrich, USA. Additionally, Mueller Hinton Broth, used for antimicrobial susceptibility testing, was supplied by Oxoid, UK. Staining and fixation procedures involved several reagents, including Crystal Violet from Sigma-Aldrich, USA, for cell viability assessments. Sodium acetate, also from Sigma-Aldrich, was used for fixing biofilms. For electron microscopy and other microscopic examinations, Glutaraldehyde at 2.5% concentration was purchased from Sigma-Aldrich, USA. For chemical assays such as antioxidant activity, DPPH radicals were obtained from Sigma-Aldrich, USA, and used to measure radical scavenging activity. Analytical-grade ascorbic and gallic acid were from Al-Gomhoria, Chemicals company. Phosphate Buffered Saline (PBS), used for washing and other laboratory procedures, was supplied by Sigma-Aldrich, USA.

### Microbiology tests

Aerobic bacterial count was carried out by streaking 0.1 (mL of each of sufficient dilution of each food sample onto plate count nutrient agar medium (Oxoid; CM325)^49^ then incubated for 48 h at 37 °C. The following formula was used to count and compute the number of colony forming units per gram of sample:

1 $$\text{Total aerobic bacterial}\text{count}/\text{g }=\text{ No}.\text{ of colonies x dilution factor}.$$

### Isolation and enumeration of *E. coli*

Carried out by streaking 0.1 mL of each of sufficient dilution of each food sample onto plates of Eosin-methylene blue agar (Oxoid; CM0069) and incubated at 37 °C for 24 h^49^.

### Isolation and enumeration of *P. aeruginosa*

M-PA-C agar medium^50^ was prepared then sterilized by boiling for 1 min and poured in plates. Spread plating 0.1 mL aliquots of the appropriate dilutions of sample (dilution usually 10^–1^ to 10^–7^) onto the medium and incubate at 41.5 °C /72 h. These colonies were confirmed by streaking on cetrimide agar plates^51^. (Oxoid; CM559), a selective medium which inhibits bacterial growth except.

### Isolation and enumeration of *S. aureus*

Was carried out by spreading 0.1 mL of each sufficient dilution onto the surface agar. Baird parker medium (Oxoid; CM0275)^52^ supplemented with egg yolk and potassium tellurite solution. Plates were incubated at 37 °C for 48 h. Suspected *S. aureus* were isolated by culturing on Brain heart infusion agar^53^ (Oxoid;CM225) slants medium for further identification.

### Detection of free coagulase *S. aureus* isolates

By slide coagulase test^53^, 100 µl of Brain heart infusion broth culture of isolated *S. aureus* was added to 0.5 mL of fivefold dilution fresh citrated rabbit plasma. The tubes were incubated at 37 °C and examined for clotting after 1, 2, 3, and 24 h.

### *B. cereus* isolation and enumeration

*B. cereus* was isolated by the surface plating technique onto the *B. cereus* agar, M833 (Oxoid; CM0617) supplemented with polymyxin B and egg yolk ^52^.

### Purification and identification of bacterial isolates

Bacterial colonies obtained from all previously mentioned media were chosen and picked up according to variation in culture characteristics and colony formation then purified by streak-plate method on Nutrient agar medium. Pure isolates were maintained on slants of the same medium at 4 °C for subsequent identification. Almost all microscopic examinations and biochemical testing used for identification were carried out according to microbial identification was done by VITEK 2 system for identification were carried out according to ^53,54^.

### Morphological and growth culture characters

Texture and pigmentation production, gram stain reaction, spore stain and capsule stain with Hiss staining technique was used to detect the presence of capsules.

### Antibiotic sensitivity test

Vitek 2 system was used to assess the antibiotic sensitivity as illustrated by ^53,54^. The VITEK 2 system is an automated tool used in clinical laboratories for microbial identification and antimicrobial susceptibility testing. Samples were inoculated into VITEK 2 test cards. Each test card contains a series of various antibiotics against the tested microorganism as follows: Cefaclor, Cefotaxime, Cefoperazone, Cefepime, Clindamycin, Imipenem, Doxycycline, Levofloxacin, Ciprofloxacin, Amikacin, Sulfamethoxazole/Trimethoprim, Azithromycin, Ampicillin, Amoxicillin/Clavulanic acid, Piperacillin/Tazobactam and Nitrofurantoin. The inoculated cards are placed into the VITEK 2 instrument, which automatically incubates them at 37 °C. After the incubation period, the growth was checked using spectrophotometer instrument to monitor the colors and turbidity changes in the wells of each test card. This reflects the biochemical reactions occurring with microorganisms. After the detection, the system analyzes the obtained data and compares it against a built-in database that contains information on the metabolic profiles of various microorganisms. Based on this analysis, VITEK 2 generates an identification report, providing information about the organism’s identity, often within a few hours.

### Collection of *R. officinalis*

The *R. officinalis* medicinal plants were obtained from farm of Plant Dep. Fac. of Science, Fayoum university. The dried leaves were ground to fine powders by electric grinder.

### Preparation aqueous rosemary leaves extract (ARLE)

100 g of rosemary leaf powdered plant material were mixed with one liter of sterile distilled water (1:10 w/v) for aqueous extraction and the mixture were kept at room temperature for 24 h with continuous mixing by magnetic stirrer. ARLE was dried until a constant dry weight. The ARLE was sterilized by (0.22 μm) Millipore filter and stored at 4 °C for further use^55^.

### Gas chromatography mass (GC–MS)

Ethanol rosemary leaves extract was prepared as follows; powdered rosemary leaves (50 g) soaked into 70% ethanol as 1:5 volumes. The mixture was kept for 24 h in tightly sealed vessels at room temperature^56^ and subjected to rotary evaporation in order to remove the ethanol and the residuals. The GC–MS analysis of the extract were performed using Perkin Elmer system (GC clarus 600, USA) equipped with an AOC-20i autosampler and gas chromatograph interfaced to a mass spectrometer (GC–MS) instrument employed in The Regional Center for Mycology and Biotechnology (RCMB), Al-Azhar Univ.

### Bioactivity of ARLE by disc diffusion assay

Impregnated paper discs with ARLE in concentration (100 mg/mL) were placed on the surface of inoculated agar plates for 24 h (~ 4 mm thickness agar layer) and incubated at 37 °C for 24 h. Negative control were prepared using sterile distilled water instead of active compounds. The susceptibility of the bacteria to each extract was estimated by measuring the diameter of the zones of inhibition and recorded values as the average of three replicates^57^.

### Mean growth inhibition by ELISA reader

It was determined by broth microdilution method. The tested bacteria suspension equivalent to the turbidity of 0.5 McFarland standard (10^8^ CFU/ml) in Mueller Hinton Broth (MHB) prepared from a fresh subculture of tested bacteria MHB by broth microdilution method. The isolated bacteria (100 µl) were added to each well of sterile 96-well flat-bottomed microtiter plate containing 100 mg/mL of ARLE. Three wells containing microbial suspension without ARLE used as control and two wells containing only medium as (background control). Optical density was measured at (620 nm) after 24 h at 37 °C using ELISA reader. Ampicillin and Gentamicin were used as standard antibiotics for gram positive (G + ^VE^) and gram negative (G-^VE^) bacteria, respectively. The percentage of bacterial growth reduction (GR %) was estimated using as reference the control treatment (without extract) as:

2 $$GR\% = \frac{C - T}{{Cx100}}$$

where, *C* = concentrations the control treatment and *T* = the concentrations under the extract treatment. The results were recorded as means ± SE of the triplicate experiment^58^.

### Minimum inhibitory concentration (MIC) assessment of ARLE

MICs Were determined by the broth microdilution method as approved by the guidelines of Clinical and Laboratory Standards Institute ^59–61^. A 100 mg/mL of ARLE using sterile distilled water. The microtiter plates were prepared by adding (100 μL) of MHB medium and 100 μl of each ARLE with various concentrations (100, 50, 25, 12.5, 6.25, 3.125, 1.562, 0.781, 0.390, 0.195 and 0.097 mg/mL. 20μL of bacterial suspension (0.5 McFarland standards was added to each of the wells except the control wells (contained broth only). An automatic ELISA reader (Sun Rise–TECAN, Inc. ®, USA) adjusted at (600 nm) to measure the absorbance of the plates incubated for 24 h at 37 °C. The minimal concentration of RLE resulting in inhibition of bacterial growth was recorded as the MIC ^62–64^.

### Antioxidant activity assay by DPPH radical scavenging activity

The antioxidant activity of ARLE was evaluated in triplicate using the DPPH for free radical scavenging at the Regional Center for Mycology and Biotechnology (RCMB) et al.-Azhar University. The average results were considered. freshly prepared methanol solution of 2,2-diphenyl-1-picrylhydrazyl (DPPH) radical at 0.004% (w/v) maintained at 10 °C. An aliquot of 40 µL RLE was prepared and added to 3 (ml) of DPPH solution. To immediately record the absorbance rate, a UV–visible spectrophotometer (Milton Roy, Spintronic 1201) was employed. We continuously tested the absorbance at 515 nm, recording data every minute until the absorbance stabilized (16 min). The amount of the reference chemical ascorbic acid and the DPPH radical absorbance without antioxidant (control) were also ascertained. We averaged the results after making three copies of each determination. The following formula was used to determine the DPPH radical’s percentage inhibition (PI):

3 $$\text{PI}= [\{(\text{AC}-\text{AT})/\text{AC}\}\text{ x }100]$$

where AC = Absorbance of control at t = 0 min and AT = absorbance of the sample + DPPH at t = 16 min^65,66^.

### Antivirulence activity

#### Assessment of biofilm formation

Biofilm formation of bacterial isolates was assayed by using tissue culture plate method as described by^3,67^. The bacteria isolates were infused with 10 ml of trypticase soy broth containing 2% glucose and allowed to incubate for a full day at 37 °C. The diluted cultures with 0.5 McFarland standard were placed in individual wells on tissue culture plates (Sigma-Aldrich, Costar, USA) containing 200 μL of each. Using 200 μL of sterile broth medium, negative control wells were infected. Incubation of the plates lasted for 24 h at 37 °C. Following incubation, each well’s contents—floating bacteria—were gently tapped out. The wells underwent four washings with 0.2 ml of phosphate buffer saline (pH = 7.2). Bacteria that adhered to the wells generated biofilm, which was fixed with 2% sodium acetate and stained with 0.1% crystal violet. Deionized water was used to remove any remaining discoloration, and the plates were left to dry. The micro ELISA microtiter-plate reader (Sun Rise–TECAN, Inc. ®, USA) was used to measure the optical density (O.D.) of the stained adherent biofilm at a wavelength of 570 nm. There were three duplicates of the experiment run. Utilizing the standards recommended by ^68^.

### Biofilm inhibition assay using tissue culture plate method

The biofilm inhibition assay was conducted using a standard tissue culture plate method. Initially, serial two-fold dilutions of the plant extract were prepared in Trypticase Soja Broth with 2% glucose (TSBGlc) to form different concentrations of 3.00, 1.50, 0.750, 0.375, and 0.184 mg/mL. Bacterial suspensions, standardized to 5 × 10^^5^ CFU/mL, were then added to each well in 50 μL aliquots. Each test included both a negative control, consisting of TSBGlc without bacterial inoculum, and a positive control containing inoculated TSBGlc without plant extract. The microtiter plates were incubated at 37 °C for 24 h to allow biofilm formation in the presence or absence of the extract. After incubation, the wells were carefully rinsed with phosphate-buffered saline (PBS) to remove non-adherent cells. The remaining biofilms were stained with crystal violet, and excess stain was rinsed off. The stained biofilms were then solubilized, and the absorbance was measured at 570 nm using a microplate reader. The optical density readings provided quantitative data on biofilm formation, allowing for comparison between treated and control samples to assess the inhibitory effect of the plant extract on biofilm development ^69^.

### Assay of anti-hemolysis for human red blood cells by ARLE

The effectiveness of ARLE against human red blood cell lysis was assessed according to ^70^. A suspension of* S. aureus* cells (10^6^/mL) was diluted 1:100 and cultivated in TSB mixed with ARLE at sub MICs of 0.781, 0.390, 0.195, and 0.097 mg/mL. As a positive control, the cells were left untreated and shaken at 250 rpm for 16 h at 37 °C in a shaking incubator. After adding 50 µL of the cell supernatant to 3% diluted human red blood cells in PBS buffer, the mixture was incubated for an hour at 37 °C with 250 rpm shaking. Centrifugation at 16,600 xg for 10 min was used to collect the supernatant, and the optical density at 540 nm was used to measure the hemolytic activity.

### Evaluation of ARLE anticancer activity

Three tumor cell lines—MCF-7 (human breast adenocarcinoma cell line), HepG-2 (human hepatocellular carcinoma cell line), and HCT-116 (human colon carcinoma cell line)—originating from the American Type Culture Collection (ATCC) continuous cell line were tested for any cytotoxic activity. The cell lines from passage number 76 are cultured and maintained in Dulbecco’s Modified Eagle’s Medium (DMEM) supplemented with 10% fetal calf serum, Hanks salt base, and 50 (µg/mL) Gentamycin antibiotic solution. Next, 96 tissue culture plates were filled with 50 (µl/well) of ARLE. An additional set of wells was reserved for the inclusion of cell control wells. These wells contained 50 µL of DMEM supplemented with 2% fetal bovine serum (FBS) in place of ARLE, which served as the negative control. A 96-well sterile tissue culture plate was filled with serial threefold dilutions of the ARLE using a multichannel pipette (Eppendorf, Germany). After applying a plate sealer to the treated and untreated cells, the plate was subsequently incubated for 24 h at 37 °C in a humidified environment containing 5% CO_2_ to enable the cells to develop and multiply. Using an inverted microscope, the plate was inspected following incubation^71^.

### Determination of cells viability

The crystal violet staining method was used to count the number of surviving cells. To remove the media, the plate holding the treated and untreated cells was either inverted or aspirated after the incubation time ended. After washing the wells with 100 µL of PBS, the cells were fixed with 10% formalin for 15 min at room temperature. The cells were then stained for 20 min with 100 µL of crystal violet, and any excess stain was removed before the plates were dried and rinsed with deionized water. The morphological alterations in treated cells relative to control cells were captured in photos by employing an inverted microscope and digital camera to investigate cellular morphology. After adding glacial acetic acid (33%), to each well, and mixing the contents, the dye was removed from the cells to provide quantitative data. The absorbance was then measured at 490 nm using an ELISA reader (Sun Rise TECAN, Inc.®, USA). As more surviving cells are added to the culture plate, the absorbance increases ^72^. Percentage cell viability was calculated as follows:

4 $$\text{\%Cell viability} = \text{(Mean Abs control}-\text{Mean Abs plant extract)}/\text{(Mean Abs control)}  x  100$$

where: Abs absorbance at 490 nm.

### Investigation of treated and non-treated bacteria

ARLE is applied to *P. aeruginosa* and* S. aureus* cultures at 6.25 and 1.562 mg/mL MICs, respectively, and the cultures are then incubated for a full day^73^. An automated tissue processor (Leica EM TP) was used to fixate the treated and untreated bacteria (as a control) with 2.5% glutaraldehyde and dehydrate them by ethanol serial dilution. After that, a CO_2_ critical point drier (Tousimis Audosamdri-815) was used to dry it. At the Regional Center of Mycology and Biotechnology in Cairo, Egypt, the bacteria growth was coated using a gold sputter coater (SPI-Module) and examined using a high vacuum mode within a JEOL JEM-1010 transmission electron microscope.

### Application of ARLE on meat preservation

From a nearby retail store, fresh sliced beef was bought. The sample of beef was sliced into four sets of tiny pieces (2.1 × 2.5 × 1 cm). The first set, ARLE was not added, which served as positive control. The second set was slices inoculated with *S. aureus* and *P. aeruginosa* into beef involved preparing bacterial suspensions at a cell concentration of 10^^6^ CFU/mL. The beef samples were aseptically placed into sterile stomacher bags, and a measured volume of each bacterial suspension was directly deposited onto the surface of individual meat pieces. The inoculation was followed by thorough homogenization, to ensure an even distribution of bacteria throughout the meat surface. This method provided controlled contamination levels and uniform bacterial colonization, enabling subsequent assessment of microbial growth and the antimicrobial efficacy of ARLE. The two another sets were treated with ARLE by thoroughly mixing meat samples at concentrations of 6.25 and 12.5 (mg/mL). Meat samples were put in plastic-film-covered petri dishes and kept at 4 °C. The bacteriological quality of each sample was checked every few days (0, 3, 6, 9, 12, 18 and 21 days) ^73^. Samples were taken aseptically on the sampling days throughout the storage of the beef cuts. Additionally, an aseptic transfer of a 10-g beef sample to a sterile stomacher bag, 90 mL of sterile Ringer solution, and two minutes of room temperature homogenization were also performed. To obtain decimal dilutions in Ringer solution, 0.1 mL samples of the corresponding dilutions were distributed or poured onto plate count agar (Oxoid; CM325) for TVC. The samples were then incubated for 48 h at 35 °C. The storage trials for the beef slices were carried out in triplicate, and the microbiota of each treatment was examined at each stage.

### Sensory properties evaluation of beef meat treated with ARLE

A sensory parameters study was conducted to assess the overall acceptability, look, texture, taste, and scent of beef meat treated with ARLE. Ten members of the panel of graduate judges, post-graduate students, staff, and families in Egypt conducted the evaluation. They were five females and five males, ranging in age from 16 to 60. They used a nine-point hedonic scale from 0 (lowest) to 9 (highest), and they followed ^73^.

### Statistical analysis

In triplicate, experimental values were analyzed using analysis of variance (ANOVA) throughout the IBM® SPSS® Statistics software (version 19) and represented as the mean ± standard division of the mean^17^. The IC_50_ values were calculated using GraphPad Prism Software v.8.4.1 (San Diego, CA, www.graphpad.com) as mean ± SD (n = 3). All charts analyzed in this study were plotted using Originpro 2022 (64-bit) SR1 v9.9.0.2 software https://www.originlab.com/index.aspx?go=Support&pid=%204440. 

## Conclusion

The study analyzed the bacterial count in beef burger and luncheon samples, revealing that gram negative bacteria had the highest food contamination rate (58%). Four selected bacteria isolates were identified based on their morphological and cultural characteristics, confirmed by VITEK 2 system analysis. *B. cereus*, *E. coli, P. earuginosa*, and *S. aureus* were identified as bacterial isolates from the Bacillaceae, Enterobacteriaceae, Pseudomonadaceae, and Staphylococcaceae families. The study found nine active compounds in rosemary leaves extract, including Ferruginol, Camphor, Cineole, Verbenone, Borneol, αcaryophyllene, Terpinen-4-ol, 2-methyl-4-vinylphenol, and Eugenol, which act as antioxidants and antibacterials. Aqueous rosemary leaves extract (ARLE) showed efficacy in antibacterial activity against various bacteria, with clear inhibition zones (CIZ) and mean growth inhibition (MGI) values of 28.1 mm and 37% respectively. The addition of ARLE to meat beef significantly reduced pathogenic bacteria during storage. The study assessed the impact of aqueous ARLE on meat beef sensory quality, finding that it enhanced the meat’s sensory properties but did not affect overall acceptability. ARLE antioxidant activity was found to be effective in food preservation as a natural antioxidant agent.

Based on the microbial analyses conducted in this study, the application of ARLE significantly extended the shelf-life of beef meat by maintaining microbial loads within acceptable safety and spoilage thresholds. Specifically, while untreated samples exceeded the spoilage limit of 10^^6^ CFU/g and safe pathogen levels within a few days, the treated samples remained below these thresholds for up to 12 days of storage. This indicates that ARLE effectively inhibited microbial growth, thereby prolonging the meat’s freshness and safety. The findings demonstrate that incorporating rosemary extract not only enhances the antimicrobial quality of meat products but also provides a quantifiable benefit in extending shelf-life to meet established microbial standards, supporting its potential as a natural preservative in meat preservation strategies.

## Supplementary Information

Supplementary Information.

## Data Availability

All data generated or analyzed during this study are included in this published article.
