# Supplementary material for: Biological activities and application of Rosmarinus officinalis extract to improve the preservation and microbial qualities of some local meat products
Source: Sci Rep. 2025 Aug 21;15:30806. doi: 10.1038/s41598-025-14247-x (PMC12371085; doi:10.1038/s41598-025-14247-x)
Supplement: Supplementary file 1 — Supplementary Information. [file 41598_2025_14247_MOESM1_ESM.pdf]

Figures list

Figure legends

Fig. (1). TEM images showing morphologically damaged cells of *Ps. aeruginosa* and *Staph. aureus* treated with MRLE (B, C, E & F) and images of morphological normal Non-treated cells (A & D).

Fig. (2). Curve of antioxidant activity of as determined by free radical-scavenging activity.

Fig. (3): Curves showing the dose response of Tumor cell line (MCF-7 , HepG2 and HCT-116) after exposure to aqueous rosemary leave extract for 24h.

Fig. (4) Photo images, showing effect of ARLE at different conc. (25, 50 and 100 ug) for 24h on HepG-2, MCF-7 and HCT-116 cell-lines comparable to control.

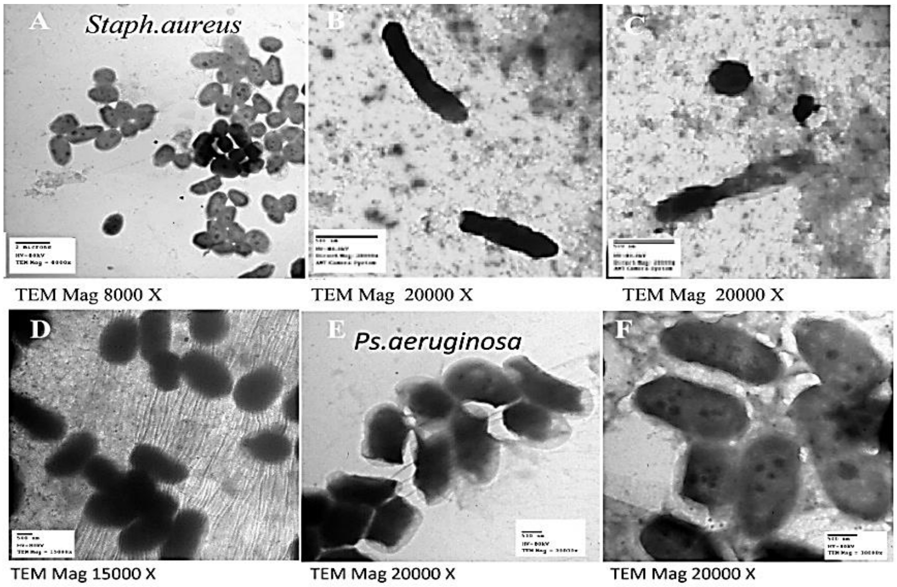
A
Staph.aureus
B
C
TEM Mag 8000 X
TEM Mag 20000 X
TEM Mag 20000 X
D
E
Ps.aeruginosa
F
TEM Mag 15000 X
TEM Mag 20000 X
TEM Mag 20000 X

Fig. 1

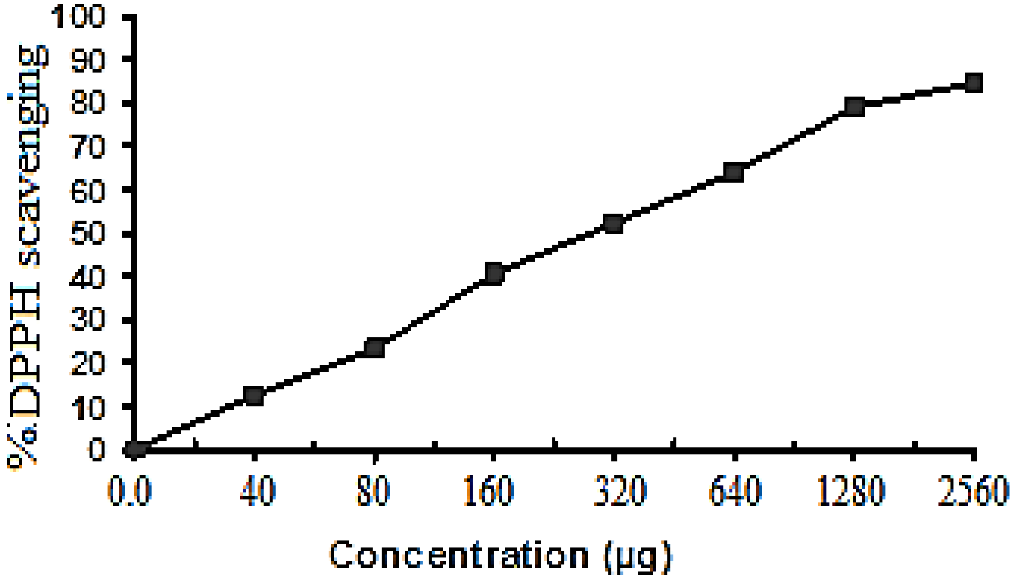
%DPPH scavenging
100
90
80
70
60
50
40
30
20
10
0
0.0
40
80
160
320
640
1280
2560
Concentration (µg)

Fig. 2

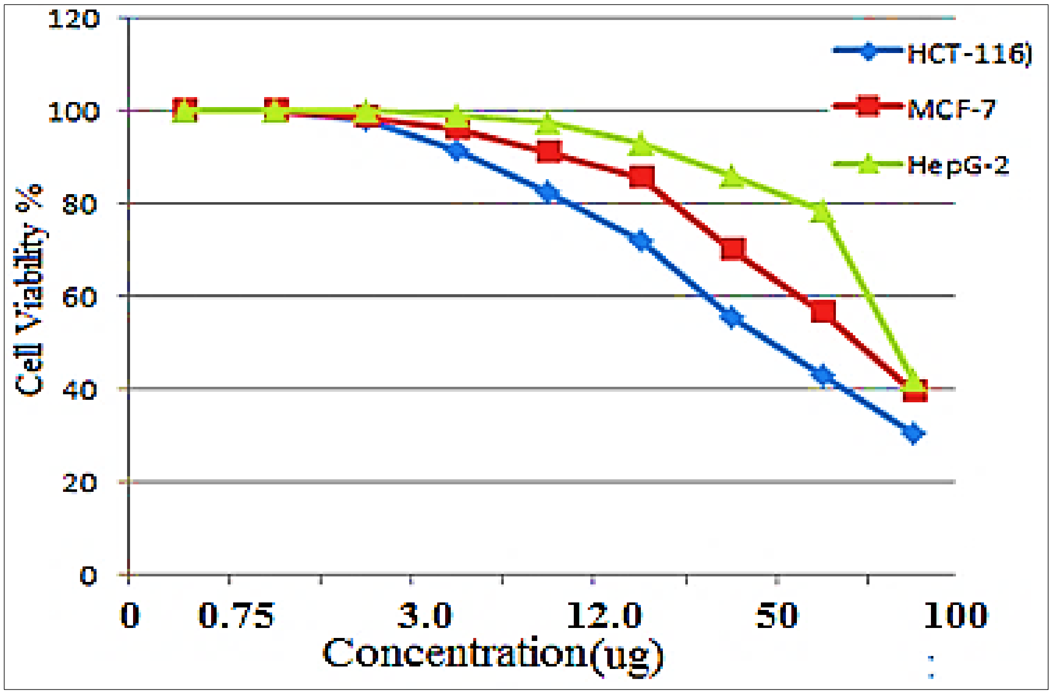
120
100
80
60
40
20
0
Cell Viability %
HCT-116)
MCF-7
HepG-2
0
0.75
3.0
12.0
50
100
Concentration(ug)

Fig. 3

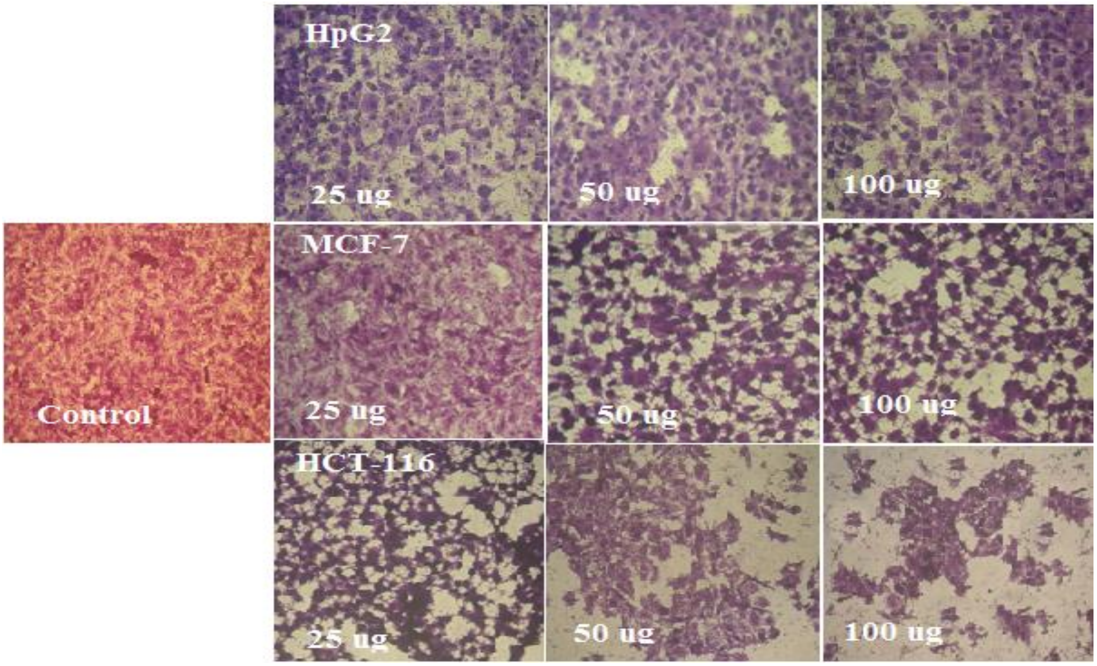
HpG2
25 ug
50 ug
100 ug
Control
MCF-7
25 ug
50 ug
100 ug
HCT-116
25 ug
50 ug
100 ug

Fig. 4
